# (*SP*-4-4)-[Hydrogen *N*-({2-[(2*S*)-1-benzyl­pyrrolidine-2-carboxamido]phen­yl}(phen­yl)methyl­ene)-l-glutamato(2−)]nickel(II)

**DOI:** 10.1107/S1600536809010861

**Published:** 2009-03-28

**Authors:** Jia-Dong Zhou, Fei Cao, Han-Jie Ying, Ping Wei

**Affiliations:** a College of Life Science and Pharmaceutical Engineering, Nanjing University of Technology, Xinmofan Road No. 5 Nanjing, Nanjing 210009, People’s Republic of China

## Abstract

In the mol­ecule of the title complex, [Ni(C_30_H_29_N_3_O_5_)], the Ni atom is coordinated in a distorted square-planar geometry by three N and one O atoms. The aromatic rings are oriented at dihedral angles of 29.01 (3), 79.73 (3) and 83.37 (3)°. The remaining rings adopt envelope conformations with the C and N atoms at the flap positions. In the crystal structure, inter­molecular O—H⋯O hydrogen bonds link the mol­ecules into chains along the *b* axis. There is also a weak C—H⋯π inter­action.

## Related literature

For the stoichiometric asymmetric synthesis of amino acids based on use of the chiral auxiliary (*S*)-2-[*N*-(*N*′-benzyl­prol­yl)amino]benzophenone, see: Belokon (1992 ▶). For non-proteinogenic amino acids synthesized by this method, see: Belokon *et al.* (1985 ▶, 1986 ▶, 1990 ▶); Belokon, Bakhmutov *et al.* (1988 ▶); Belokon, Bulychev *et al.* (1988 ▶); Belokon, Sagyan *et al.* (1988 ▶); Soloshonok *et al.* (1992 ▶, 2001 ▶). For bond-length data, see: Allen *et al.* (1987 ▶).
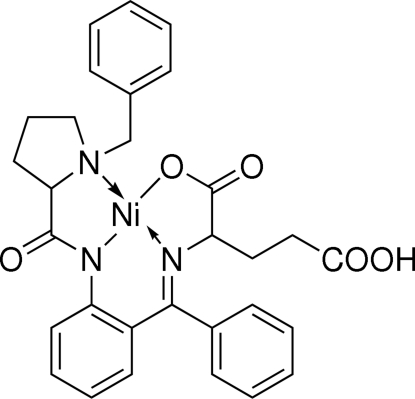


## Experimental

### 

#### Crystal data

[Ni(C_30_H_29_N_3_O_5_)]
*M* *_r_* = 570.27
Orthorhombic, 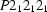

*a* = 9.4570 (19) Å
*b* = 14.293 (3) Å
*c* = 20.251 (4) Å
*V* = 2737.3 (10) Å^3^
*Z* = 4
Mo *K*α radiation
μ = 0.75 mm^−1^
*T* = 294 K
0.30 × 0.20 × 0.20 mm

#### Data collection

Nonius–Nonius CAD-4 diffractometer
Absorption correction: ψ scan (North *et al.*, 1968 ▶) *T*_min_ = 0.806, *T*_max_ = 0.864
5494 measured reflections
4976 independent reflections
4168 reflections with *I* > 2σ(*I*)
*R*_int_ = 0.067
3 standard reflections frequency: 120 min intensity decay: 1%

#### Refinement

*R*[*F* ^2^ > 2σ(*F* ^2^)] = 0.047
*wR*(*F* ^2^) = 0.143
*S* = 1.00
4976 reflections
334 parameters
H-atom parameters constrained
Δρ_max_ = 0.39 e Å^−3^
Δρ_min_ = −0.48 e Å^−3^
Absolute structure: Flack (1983 ▶), 2145 Friedel pairs
Flack parameter: 0.00 (2)

### 

Data collection: *CAD-4 Software* (Enraf–Nonius, 1989 ▶); cell refinement: *CAD-4 Software*; data reduction: *XCAD4* (Harms & Wocadlo, 1995 ▶); program(s) used to solve structure: *SHELXS97* (Sheldrick, 2008 ▶); program(s) used to refine structure: *SHELXL97* (Sheldrick, 2008 ▶); molecular graphics: *PLATON* (Spek, 2009 ▶); software used to prepare material for publication: *SHELXL97* .

## Supplementary Material

Crystal structure: contains datablocks global, I. DOI: 10.1107/S1600536809010861/hk2646sup1.cif

Structure factors: contains datablocks I. DOI: 10.1107/S1600536809010861/hk2646Isup2.hkl

Additional supplementary materials:  crystallographic information; 3D view; checkCIF report

## Figures and Tables

**Table:** 

Ni—N2	1.843 (4)
Ni—O5	1.850 (3)
Ni—N3	1.859 (4)
Ni—N1	1.931 (4)

**Table:** 

N2—Ni—O5	174.69 (17)
N2—Ni—N3	95.59 (16)
O5—Ni—N3	86.41 (15)
N2—Ni—N1	87.02 (16)
O5—Ni—N1	91.78 (15)
N3—Ni—N1	170.73 (18)

**Table 2:** Hydrogen-bond geometry (Å, °)

*D*—H⋯*A*	*D*—H	H⋯*A*	*D*⋯*A*	*D*—H⋯*A*
O3—H3*B*⋯O4^i^	0.82	1.86	2.680 (6)	174
C2—H2*A*⋯*Cg*1^ii^	0.93	2.93	3.722 (5)	144

Symmetry codes: (i) 
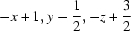
; (ii) 
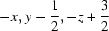
. *Cg*1 is the centroid of the C13–C18 ring.

**Fig. 1.**
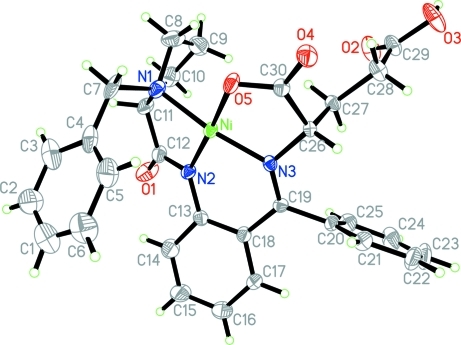
The molecular structure of the title molecule, with the atom-numbering scheme. Displacement ellipsoids are drawn at the 30% probability level.

**Fig. 2.**
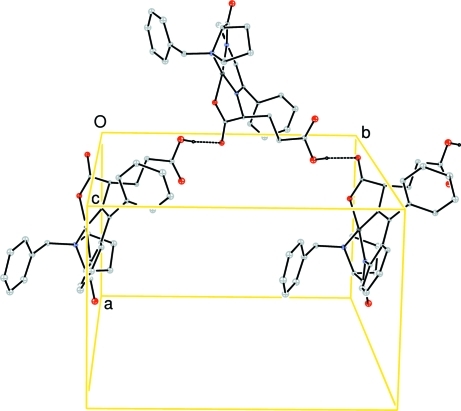
A partial packing diagram of the title compound. Hydrogen bonds are shown as dashed lines. Hydrogen atoms not involved in hydrogen bonding have been omitted.

## supplementary crystallographic information

### Comment

The method of stoichiometric asymmetric synthesis of amino acids based on use of the chiral auxiliary (S)-2-[N-(N'-benzylprolyl)amino]benzophenone (BPB) developed by Belokon's group (Belokon, 1992) was one of the most versatile and general methods for amino acids preparations. With inexpensive reagents and simple experimental procedures, many kinds of tailor-made non-proteinogenic amino acids were synthesized by this method (Belokon *et al.*, 1985; Belokon *et al.*,  1986; Belokon, Bakhmutov *et al.*, 1988; Belokon, Bulychev *et al.*, 1988; Belokon, Sagyan *et al.*, 1988; Belokon *et al.*,  1990; Soloshonok *et al.*,  1992; Soloshonok *et al.*,  2001). Here we applied this method for the synthesis of gamma-L-glutamyl dipeptides via a Ni complex of glutamic acid Schiff base (the title complex), which has been employed as co-protection of the alpha-amino and alpha-carboxyl group of L-glutamic acid. Then, the title complex reacted with L-amino acids to give Ni complexes of gamma-L-glutamyl dipeptides Schiff base. Finally, after decomposition of these complexes, gamma-L-glutamyl dipeptides were obtained and the chiral auxiliary BPB were recovered in high yields. We report herein the crystal structure of the title complex.

In the title complex, (Fig. 1), the Ni atom is in a distorted square-planar coordination by three N and one O atoms. The Ni-N and Ni-O bond lengths (Allen *et al.*,  1987) and angles (Table 1) are within normal ranges. Rings A (C1-C6), B (C13-C18) and C (C20-C25) are, of course, planar and they are oriented at dihedral angles of A/B = 29.01 (3), A/C = 79.73 (3) and B/C = 83.37 (3) °. Rings D (N1/C8-C11), E (Ni/N1/N2/C11/C12), F (Ni/N2/N3/C13/C18/C19) and G (Ni/O5/N3/C26/C30) adopt envelope conformations with atoms C8, N1, C19 and N3 displaced by 0.609 (3), -0.431 (3), 0.421 (3) and -0.443 (3) Å, respectively, from the planes of the other ring atoms.

In the crystal structure, intermolecular O-H···O hydrogen bonds (Table 2) link the molecules into chains along the b-axis, in which they may be effective in the stabilization of the structure. There is also a weak C—H···π interaction (Table 2).

### Experimental

For the preparation of the title complex, a solution of KOH (4.49 g, 0.08 mol) in MeOH (15 ml) was poured into a mechanically stirred mixture of BPB (1.92 g, 0.005 mol), nickel chloride hexahydrate (2.38 g, 0.025 mol) and L-glutamic acid (3.68 g, 0.025 mol) in MeOH (17.5 ml) under argon atmosphere at 313-323 K. The resulting mixture was stirred at 328-338 K for 2 h, and then neutralized with AcOH (4.6 ml, 0.08 mol) and diluted with water (200 ml). After 6 h, the separated crystalline solid was filtered and washed twice with water. The title complex was purified by recrystallization in an acetone solution. Crystals suitable for X-ray analysis were obtained by slow evaporation of an acetone/hexane/AcOH (6:4:1) mixture after three weeks.

### Refinement

H atoms were positioned geometrically, with O-H = 0.82 Å (for OH) and C-H = 0.93, 0.98 and 0.97 Å for aromatic, methine and methylene H and constrained to ride on their parent atoms, with U_iso_(H) = xU_eq_(C,O), where x = 1.5 for OH H and x = 1.2 for all other H atoms.

### Crystal data

**Table:**

[Ni(C_30_H_29_N_3_O_5_)]	*F*(000) = 1192
*M**_r_* = 570.27	*D*_x_ = 1.384 Mg m^−^^3^
Orthorhombic, *P*2_1_2_1_2_1_	Mo *K*α radiation, λ = 0.71073 Å
Hall symbol: P 2ac 2ab	Cell parameters from 25 reflections
*a* = 9.4570 (19) Å	θ = 9–14°
*b* = 14.293 (3) Å	µ = 0.75 mm^−^^1^
*c* = 20.251 (4) Å	*T* = 294 K
*V* = 2737.3 (10) Å^3^	Block, red
*Z* = 4	0.30 × 0.20 × 0.20 mm

### Data collection

**Table:**

Nonius–Nonius CAD-4 diffractometer	4168 reflections with *I* > 2σ(*I*)
Radiation source: fine-focus sealed tube	*R*_int_ = 0.067
graphite	θ_max_ = 25.3°, θ_min_ = 1.7°
ω/2θ scans	*h* = 0→11
Absorption correction: ψ scan (North *et al.*, 1968)	*k* = 0→17
*T*_min_ = 0.806, *T*_max_ = 0.864	*l* = −24→24
5494 measured reflections	3 standard reflections every 120 min
4976 independent reflections	intensity decay: 1%

### Refinement

**Table:**

Refinement on *F*^2^	Hydrogen site location: inferred from neighbouring sites
Least-squares matrix: full	H-atom parameters constrained
*R*[*F*^2^ > 2σ(*F*^2^)] = 0.047	*w* = 1/[σ^2^(*F*_o_^2^) + (0.1*P*)^2^ + 0.28*P*] where *P* = (*F*_o_^2^ + 2*F*_c_^2^)/3
*wR*(*F*^2^) = 0.143	(Δ/σ)_max_ = 0.001
*S* = 1.00	Δρ_max_ = 0.39 e Å^−^^3^
4976 reflections	Δρ_min_ = −0.48 e Å^−^^3^
334 parameters	Absolute structure: Flack (1983), 2145 Friedel pairs
Primary atom site location: structure-invariant direct methods	Flack parameter: 0.00 (2)
Secondary atom site location: difference Fourier map	

### Special details

**Table:**

Geometry. All e.s.d.'s (except the e.s.d. in the dihedral angle between two l.s. planes) are estimated using the full covariance matrix. The cell e.s.d.'s are taken into account individually in the estimation of e.s.d.'s in distances, angles and torsion angles; correlations between e.s.d.'s in cell parameters are only used when they are defined by crystal symmetry. An approximate (isotropic) treatment of cell e.s.d.'s is used for estimating e.s.d.'s involving l.s. planes.
Refinement. Refinement of *F*^2^ against ALL reflections. The weighted *R*-factor *wR* and goodness of fit *S* are based on *F*^2^, conventional *R*-factors *R* are based on *F*, with *F* set to zero for negative *F*^2^. The threshold expression of *F*^2^ > σ(*F*^2^) is used only for calculating *R*-factors(gt) *etc*. and is not relevant to the choice of reflections for refinement. *R*-factors based on *F*^2^ are statistically about twice as large as those based on *F*, and *R*- factors based on ALL data will be even larger.

### Fractional atomic coordinates and isotropic or equivalent isotropic displacement parameters (Å^2^)

**Table:**

	*x*	*y*	*z*	*U*_iso_*/*U*_eq_	
Ni	0.10306 (6)	0.03868 (4)	0.87481 (3)	0.02921 (16)	
O1	−0.2907 (4)	0.0036 (3)	0.9260 (2)	0.0672 (13)	
O2	0.3316 (4)	−0.3086 (3)	0.7881 (2)	0.0709 (13)	
O3	0.5596 (4)	−0.3039 (3)	0.7646 (3)	0.0745 (14)	
H3B	0.5403	−0.3513	0.7436	0.112*	
O4	0.4826 (3)	0.0397 (3)	0.80568 (16)	0.0444 (8)	
O5	0.2552 (3)	0.0681 (2)	0.82123 (17)	0.0418 (9)	
N1	−0.0300 (4)	0.0824 (3)	0.8094 (2)	0.0410 (10)	
N2	−0.0498 (4)	0.0211 (3)	0.92988 (18)	0.0332 (9)	
N3	0.2332 (4)	−0.0207 (2)	0.92937 (17)	0.0274 (8)	
C1	−0.1321 (8)	0.3251 (5)	0.9817 (5)	0.087	
H1A	−0.1574	0.3511	1.0221	0.105*	
C2	−0.2344 (9)	0.3103 (5)	0.9359 (4)	0.079	
H2A	−0.3270	0.3295	0.9436	0.095*	
C3	−0.1983 (8)	0.2664 (5)	0.8781 (5)	0.087 (2)	
H3A	−0.2677	0.2558	0.8465	0.105*	
C4	−0.0602 (6)	0.2372 (4)	0.8655 (4)	0.0598 (15)	
C5	0.0440 (8)	0.2567 (4)	0.9092 (4)	0.0727 (19)	
H5A	0.1375	0.2408	0.9004	0.087*	
C6	0.0050 (8)	0.3036 (5)	0.9709 (4)	0.080	
H6A	0.0736	0.3184	1.0021	0.096*	
C7	−0.0263 (6)	0.1871 (4)	0.8028 (3)	0.0541 (15)	
H7A	0.0671	0.2059	0.7881	0.065*	
H7B	−0.0935	0.2060	0.7691	0.065*	
C8	−0.0002 (6)	0.0406 (6)	0.7432 (2)	0.0621 (16)	
H8A	−0.0502	0.0741	0.7087	0.075*	
H8B	0.1003	0.0420	0.7337	0.075*	
C9	−0.0521 (6)	−0.0571 (5)	0.7487 (3)	0.0694 (19)	
H9A	0.0134	−0.0957	0.7736	0.083*	
H9B	−0.0669	−0.0847	0.7055	0.083*	
C10	−0.1893 (6)	−0.0450 (5)	0.7850 (3)	0.0636 (15)	
H10A	−0.2080	−0.0988	0.8128	0.076*	
H10B	−0.2669	−0.0377	0.7541	0.076*	
C11	−0.1723 (5)	0.0431 (4)	0.8270 (2)	0.0406 (11)	
H11A	−0.2463	0.0883	0.8156	0.049*	
C12	−0.1781 (5)	0.0219 (4)	0.9002 (2)	0.0434 (12)	
C13	−0.0335 (5)	0.0151 (3)	0.9983 (2)	0.0344 (10)	
C14	−0.1412 (6)	0.0480 (5)	1.0402 (3)	0.0597 (15)	
H14A	−0.2237	0.0720	1.0219	0.072*	
C15	−0.1267 (7)	0.0453 (5)	1.1074 (3)	0.0700 (19)	
H15A	−0.1974	0.0705	1.1340	0.084*	
C16	−0.0091 (7)	0.0060 (5)	1.1362 (3)	0.0649 (17)	
H16A	−0.0021	0.0014	1.1819	0.078*	
C17	0.0988 (6)	−0.0268 (4)	1.0961 (2)	0.0455 (11)	
H17A	0.1793	−0.0525	1.1152	0.055*	
C18	0.0887 (5)	−0.0217 (3)	1.0272 (2)	0.0336 (10)	
C19	0.2138 (4)	−0.0498 (3)	0.98942 (19)	0.0286 (9)	
C20	0.3174 (5)	−0.1124 (3)	1.0232 (2)	0.0325 (10)	
C21	0.4508 (6)	−0.0831 (4)	1.0417 (3)	0.0488 (13)	
H21A	0.4797	−0.0220	1.0332	0.059*	
C22	0.5418 (6)	−0.1454 (6)	1.0730 (3)	0.068 (2)	
H22A	0.6322	−0.1265	1.0851	0.082*	
C23	0.4972 (8)	−0.2360 (6)	1.0862 (3)	0.081 (2)	
H23A	0.5574	−0.2775	1.1076	0.097*	
C24	0.3652 (8)	−0.2642 (5)	1.0678 (3)	0.074 (2)	
H24A	0.3358	−0.3249	1.0769	0.088*	
C25	0.2750 (6)	−0.2036 (4)	1.0357 (3)	0.0513 (13)	
H25A	0.1859	−0.2238	1.0224	0.062*	
C26	0.3611 (4)	−0.0461 (3)	0.89063 (18)	0.0254 (8)	
H26A	0.4457	−0.0443	0.9186	0.031*	
C27	0.3408 (5)	−0.1430 (3)	0.8613 (2)	0.0330 (10)	
H27A	0.3153	−0.1861	0.8964	0.040*	
H27B	0.2629	−0.1411	0.8302	0.040*	
C28	0.4708 (5)	−0.1796 (4)	0.8265 (3)	0.0467 (13)	
H28A	0.5021	−0.1337	0.7944	0.056*	
H28B	0.5460	−0.1879	0.8585	0.056*	
C29	0.4445 (6)	−0.2708 (4)	0.7921 (3)	0.0472 (13)	
C30	0.3713 (4)	0.0262 (3)	0.8355 (2)	0.0305 (9)	

### Atomic displacement parameters (Å^2^)

**Table:**

	*U*^11^	*U*^22^	*U*^33^	*U*^12^	*U*^13^	*U*^23^
Ni	0.0254 (2)	0.0311 (3)	0.0311 (3)	0.0020 (2)	0.0025 (2)	0.0091 (2)
O1	0.0267 (18)	0.118 (4)	0.057 (2)	−0.013 (2)	0.0037 (16)	0.030 (2)
O2	0.044 (2)	0.062 (3)	0.106 (4)	0.000 (2)	−0.006 (2)	−0.044 (3)
O3	0.054 (2)	0.055 (2)	0.114 (4)	−0.0033 (19)	0.031 (2)	−0.045 (3)
O4	0.0334 (17)	0.049 (2)	0.0514 (19)	−0.0001 (17)	0.0132 (14)	0.0137 (19)
O5	0.0295 (16)	0.048 (2)	0.048 (2)	0.0069 (14)	0.0043 (14)	0.0258 (15)
N1	0.029 (2)	0.052 (3)	0.042 (2)	0.0028 (18)	0.0007 (18)	0.0144 (19)
N2	0.0316 (18)	0.038 (2)	0.0300 (18)	0.0017 (17)	0.0020 (15)	0.0074 (16)
N3	0.0294 (18)	0.0202 (19)	0.0327 (19)	0.0019 (14)	0.0035 (15)	0.0020 (15)
C1	0.080	0.070	0.112	0.006	0.011	−0.016
C2	0.079	0.079	0.079	0.000	0.000	0.000
C3	0.080 (4)	0.058 (4)	0.124 (6)	0.020 (3)	0.012 (5)	0.002 (4)
C4	0.060 (3)	0.034 (3)	0.086 (4)	0.009 (2)	0.000 (3)	0.020 (3)
C5	0.081 (4)	0.044 (3)	0.093 (5)	0.004 (3)	−0.003 (3)	0.006 (3)
C6	0.074	0.061	0.105	−0.021	−0.006	−0.011
C7	0.043 (3)	0.050 (3)	0.070 (4)	0.002 (2)	0.001 (3)	0.042 (3)
C8	0.049 (3)	0.103 (5)	0.034 (3)	−0.001 (4)	−0.004 (2)	0.001 (3)
C9	0.058 (4)	0.095 (6)	0.055 (3)	0.014 (4)	−0.011 (3)	−0.018 (4)
C10	0.056 (3)	0.071 (4)	0.064 (4)	−0.004 (3)	−0.016 (3)	−0.002 (3)
C11	0.027 (2)	0.056 (3)	0.039 (2)	−0.001 (3)	−0.0052 (18)	0.009 (3)
C12	0.037 (2)	0.047 (3)	0.046 (3)	0.008 (2)	0.006 (2)	0.013 (2)
C13	0.034 (2)	0.035 (3)	0.033 (2)	0.0022 (19)	0.0107 (19)	0.0062 (18)
C14	0.049 (3)	0.079 (4)	0.052 (3)	0.015 (3)	0.014 (2)	0.012 (3)
C15	0.062 (4)	0.110 (5)	0.038 (3)	0.023 (4)	0.016 (2)	0.004 (3)
C16	0.074 (4)	0.086 (4)	0.034 (3)	0.015 (3)	0.017 (3)	0.008 (3)
C17	0.049 (3)	0.055 (3)	0.033 (2)	0.014 (3)	−0.004 (2)	0.003 (2)
C18	0.030 (2)	0.036 (2)	0.034 (2)	0.003 (2)	0.0039 (18)	0.0050 (18)
C19	0.038 (2)	0.023 (2)	0.0248 (19)	−0.0011 (18)	0.0016 (16)	0.0050 (17)
C20	0.032 (2)	0.039 (2)	0.026 (2)	0.008 (2)	0.0026 (18)	0.0060 (19)
C21	0.047 (3)	0.059 (3)	0.041 (3)	0.008 (3)	−0.003 (2)	−0.001 (2)
C22	0.038 (3)	0.114 (6)	0.052 (3)	0.023 (4)	−0.005 (3)	0.012 (4)
C23	0.060 (4)	0.110 (6)	0.071 (4)	0.048 (4)	0.009 (3)	0.040 (4)
C24	0.089 (5)	0.058 (4)	0.074 (4)	0.036 (4)	0.022 (4)	0.033 (3)
C25	0.061 (3)	0.045 (3)	0.048 (3)	0.005 (3)	0.009 (3)	0.015 (2)
C26	0.0232 (19)	0.024 (2)	0.029 (2)	0.0026 (17)	0.0000 (14)	0.0006 (17)
C27	0.036 (2)	0.026 (2)	0.037 (3)	0.0020 (18)	0.0061 (19)	−0.0003 (18)
C28	0.037 (3)	0.042 (3)	0.061 (3)	−0.001 (2)	0.007 (2)	−0.012 (3)
C29	0.042 (3)	0.040 (3)	0.060 (3)	0.005 (2)	0.006 (2)	−0.018 (3)
C30	0.026 (2)	0.030 (2)	0.035 (2)	−0.0024 (19)	0.0024 (17)	0.0031 (18)

### Geometric parameters (Å, °)

**Table:**

Ni—N2	1.843 (4)	C10—C11	1.527 (9)
Ni—O5	1.850 (3)	C10—H10A	0.9700
Ni—N3	1.859 (4)	C10—H10B	0.9700
Ni—N1	1.931 (4)	C11—C12	1.515 (7)
O1—C12	1.215 (6)	C11—H11A	0.9800
O2—C29	1.200 (6)	C13—C18	1.398 (6)
O3—C29	1.310 (6)	C13—C14	1.407 (7)
O3—H3B	0.8200	C14—C15	1.368 (8)
O4—C30	1.229 (5)	C14—H14A	0.9300
O5—C30	1.283 (5)	C15—C16	1.376 (9)
N1—C8	1.493 (7)	C15—H15A	0.9300
N1—C11	1.501 (6)	C16—C17	1.386 (7)
N1—C7	1.504 (7)	C16—H16A	0.9300
N2—C12	1.353 (6)	C17—C18	1.400 (6)
N2—C13	1.397 (6)	C17—H17A	0.9300
N3—C19	1.298 (5)	C18—C19	1.465 (6)
N3—C26	1.487 (5)	C19—C20	1.492 (6)
C1—C6	1.351 (10)	C20—C21	1.382 (7)
C1—C2	1.357 (10)	C20—C25	1.387 (7)
C1—H1A	0.9300	C21—C22	1.392 (8)
C2—C3	1.372 (11)	C21—H21A	0.9300
C2—H2A	0.9300	C22—C23	1.386 (11)
C3—C4	1.395 (9)	C22—H22A	0.9300
C3—H3A	0.9300	C23—C24	1.364 (11)
C4—C5	1.354 (9)	C23—H23A	0.9300
C4—C7	1.492 (9)	C24—C25	1.378 (8)
C5—C6	1.466 (11)	C24—H24A	0.9300
C5—H5A	0.9300	C25—H25A	0.9300
C6—H6A	0.9300	C26—C27	1.520 (6)
C7—H7A	0.9700	C26—C30	1.524 (6)
C7—H7B	0.9700	C26—H26A	0.9800
C8—C9	1.485 (10)	C27—C28	1.511 (6)
C8—H8A	0.9700	C27—H27A	0.9700
C8—H8B	0.9700	C27—H27B	0.9700
C9—C10	1.501 (8)	C28—C29	1.499 (7)
C9—H9A	0.9700	C28—H28A	0.9700
C9—H9B	0.9700	C28—H28B	0.9700
			
N2—Ni—O5	174.69 (17)	C10—C11—H11A	109.7
N2—Ni—N3	95.59 (16)	O1—C12—N2	126.4 (4)
O5—Ni—N3	86.41 (15)	O1—C12—C11	119.7 (4)
N2—Ni—N1	87.02 (16)	N2—C12—C11	113.8 (4)
O5—Ni—N1	91.78 (15)	N2—C13—C18	122.0 (4)
N3—Ni—N1	170.73 (18)	N2—C13—C14	119.9 (4)
C29—O3—H3B	109.5	C18—C13—C14	118.1 (4)
C30—O5—Ni	115.3 (3)	C15—C14—C13	121.3 (5)
C8—N1—C11	103.5 (4)	C15—C14—H14A	119.4
C8—N1—C7	108.3 (5)	C13—C14—H14A	119.4
C11—N1—C7	114.5 (4)	C14—C15—C16	120.9 (5)
C8—N1—Ni	111.3 (3)	C14—C15—H15A	119.6
C11—N1—Ni	107.5 (3)	C16—C15—H15A	119.6
C7—N1—Ni	111.6 (3)	C15—C16—C17	119.0 (5)
C12—N2—C13	122.7 (4)	C15—C16—H16A	120.5
C12—N2—Ni	115.7 (3)	C17—C16—H16A	120.5
C13—N2—Ni	121.5 (3)	C16—C17—C18	121.1 (5)
C19—N3—C26	122.0 (4)	C16—C17—H17A	119.4
C19—N3—Ni	127.6 (3)	C18—C17—H17A	119.4
C26—N3—Ni	109.7 (2)	C13—C18—C17	119.5 (4)
C6—C1—C2	122.6 (8)	C13—C18—C19	123.5 (4)
C6—C1—H1A	118.7	C17—C18—C19	116.8 (4)
C2—C1—H1A	118.7	N3—C19—C18	121.0 (4)
C1—C2—C3	118.5 (8)	N3—C19—C20	121.9 (4)
C1—C2—H2A	120.8	C18—C19—C20	117.1 (3)
C3—C2—H2A	120.8	C21—C20—C25	120.0 (5)
C2—C3—C4	121.8 (9)	C21—C20—C19	122.8 (4)
C2—C3—H3A	119.1	C25—C20—C19	117.2 (4)
C4—C3—H3A	119.1	C20—C21—C22	119.6 (6)
C5—C4—C3	120.0 (7)	C20—C21—H21A	120.2
C5—C4—C7	120.0 (6)	C22—C21—H21A	120.2
C3—C4—C7	120.0 (7)	C23—C22—C21	119.8 (6)
C4—C5—C6	118.0 (7)	C23—C22—H22A	120.1
C4—C5—H5A	121.0	C21—C22—H22A	120.1
C6—C5—H5A	121.0	C24—C23—C22	120.1 (6)
C1—C6—C5	118.9 (8)	C24—C23—H23A	119.9
C1—C6—H6A	120.5	C22—C23—H23A	119.9
C5—C6—H6A	120.5	C23—C24—C25	120.6 (6)
C4—C7—N1	113.3 (4)	C23—C24—H24A	119.7
C4—C7—H7A	108.9	C25—C24—H24A	119.7
N1—C7—H7A	108.9	C24—C25—C20	119.8 (6)
C4—C7—H7B	108.9	C24—C25—H25A	120.1
N1—C7—H7B	108.9	C20—C25—H25A	120.1
H7A—C7—H7B	107.7	N3—C26—C27	109.0 (3)
C9—C8—N1	104.3 (5)	N3—C26—C30	105.8 (3)
C9—C8—H8A	110.9	C27—C26—C30	109.9 (3)
N1—C8—H8A	110.9	N3—C26—H26A	110.7
C9—C8—H8B	110.9	C27—C26—H26A	110.7
N1—C8—H8B	110.9	C30—C26—H26A	110.7
H8A—C8—H8B	108.9	C28—C27—C26	113.2 (4)
C8—C9—C10	102.3 (5)	C28—C27—H27A	108.9
C8—C9—H9A	111.3	C26—C27—H27A	108.9
C10—C9—H9A	111.3	C28—C27—H27B	108.9
C8—C9—H9B	111.3	C26—C27—H27B	108.9
C10—C9—H9B	111.3	H27A—C27—H27B	107.7
H9A—C9—H9B	109.2	C29—C28—C27	112.5 (4)
C9—C10—C11	106.1 (5)	C29—C28—H28A	109.1
C9—C10—H10A	110.5	C27—C28—H28A	109.1
C11—C10—H10A	110.5	C29—C28—H28B	109.1
C9—C10—H10B	110.5	C27—C28—H28B	109.1
C11—C10—H10B	110.5	H28A—C28—H28B	107.8
H10A—C10—H10B	108.7	O2—C29—O3	123.3 (5)
N1—C11—C12	109.8 (4)	O2—C29—C28	124.7 (4)
N1—C11—C10	105.7 (4)	O3—C29—C28	112.0 (5)
C12—C11—C10	112.1 (5)	O4—C30—O5	123.3 (4)
N1—C11—H11A	109.7	O4—C30—C26	121.3 (4)
C12—C11—H11A	109.7	O5—C30—C26	115.3 (3)
			
N3—Ni—O5—C30	10.8 (3)	C12—N2—C13—C18	154.4 (5)
N1—Ni—O5—C30	−160.1 (4)	Ni—N2—C13—C18	−31.0 (6)
N2—Ni—N1—C8	−134.8 (4)	C12—N2—C13—C14	−26.6 (7)
O5—Ni—N1—C8	50.4 (4)	Ni—N2—C13—C14	148.0 (4)
N2—Ni—N1—C11	−22.1 (4)	N2—C13—C14—C15	−178.2 (6)
O5—Ni—N1—C11	163.0 (4)	C18—C13—C14—C15	0.8 (9)
N2—Ni—N1—C7	104.2 (4)	C13—C14—C15—C16	−3.3 (11)
O5—Ni—N1—C7	−70.7 (3)	C14—C15—C16—C17	3.4 (11)
N3—Ni—N2—C12	−155.9 (4)	C15—C16—C17—C18	−1.0 (10)
N1—Ni—N2—C12	15.2 (4)	N2—C13—C18—C17	−179.5 (4)
N3—Ni—N2—C13	29.1 (4)	C14—C13—C18—C17	1.5 (7)
N1—Ni—N2—C13	−159.8 (4)	N2—C13—C18—C19	5.1 (7)
N2—Ni—N3—C19	−8.3 (4)	C14—C13—C18—C19	−173.9 (5)
O5—Ni—N3—C19	166.8 (4)	C16—C17—C18—C13	−1.4 (8)
N2—Ni—N3—C26	162.0 (3)	C16—C17—C18—C19	174.3 (5)
O5—Ni—N3—C26	−22.9 (3)	C26—N3—C19—C18	178.3 (4)
C6—C1—C2—C3	4.2 (13)	Ni—N3—C19—C18	−12.5 (6)
C1—C2—C3—C4	−0.1 (11)	C26—N3—C19—C20	−1.2 (6)
C2—C3—C4—C5	−3.8 (10)	Ni—N3—C19—C20	168.0 (3)
C2—C3—C4—C7	178.2 (6)	C13—C18—C19—N3	17.8 (7)
C3—C4—C5—C6	3.6 (9)	C17—C18—C19—N3	−157.7 (4)
C7—C4—C5—C6	−178.4 (5)	C13—C18—C19—C20	−162.6 (4)
C2—C1—C6—C5	−4.2 (12)	C17—C18—C19—C20	21.9 (6)
C4—C5—C6—C1	0.2 (10)	N3—C19—C20—C21	68.7 (6)
C5—C4—C7—N1	87.6 (7)	C18—C19—C20—C21	−110.9 (5)
C3—C4—C7—N1	−94.5 (7)	N3—C19—C20—C25	−111.1 (5)
C8—N1—C7—C4	177.8 (5)	C18—C19—C20—C25	69.4 (5)
C11—N1—C7—C4	63.0 (6)	C25—C20—C21—C22	−0.2 (7)
Ni—N1—C7—C4	−59.4 (5)	C19—C20—C21—C22	−180.0 (5)
C11—N1—C8—C9	−39.9 (5)	C20—C21—C22—C23	−0.8 (9)
C7—N1—C8—C9	−161.8 (4)	C21—C22—C23—C24	0.8 (10)
Ni—N1—C8—C9	75.3 (5)	C22—C23—C24—C25	0.4 (11)
N1—C8—C9—C10	42.2 (5)	C23—C24—C25—C20	−1.4 (9)
C8—C9—C10—C11	−28.2 (6)	C21—C20—C25—C24	1.3 (8)
C8—N1—C11—C12	142.5 (5)	C19—C20—C25—C24	−178.9 (5)
C7—N1—C11—C12	−99.8 (5)	C19—N3—C26—C27	81.6 (4)
Ni—N1—C11—C12	24.7 (5)	Ni—N3—C26—C27	−89.4 (3)
C8—N1—C11—C10	21.4 (5)	C19—N3—C26—C30	−160.3 (4)
C7—N1—C11—C10	139.0 (5)	Ni—N3—C26—C30	28.7 (4)
Ni—N1—C11—C10	−96.4 (4)	N3—C26—C27—C28	−174.3 (4)
C9—C10—C11—N1	4.2 (6)	C30—C26—C27—C28	70.2 (5)
C9—C10—C11—C12	−115.5 (5)	C26—C27—C28—C29	−174.2 (4)
C13—N2—C12—O1	−12.0 (9)	C27—C28—C29—O2	4.8 (9)
Ni—N2—C12—O1	173.1 (5)	C27—C28—C29—O3	−177.2 (5)
C13—N2—C12—C11	171.5 (4)	Ni—O5—C30—O4	−178.3 (4)
Ni—N2—C12—C11	−3.4 (6)	Ni—O5—C30—C26	4.6 (5)
N1—C11—C12—O1	168.4 (5)	N3—C26—C30—O4	160.9 (4)
C10—C11—C12—O1	−74.4 (7)	C27—C26—C30—O4	−81.6 (5)
N1—C11—C12—N2	−14.8 (7)	N3—C26—C30—O5	−21.9 (5)
C10—C11—C12—N2	102.4 (5)	C27—C26—C30—O5	95.6 (4)

### Hydrogen-bond geometry (Å, °)

**Table:**

*D*—H···*A*	*D*—H	H···*A*	*D*···*A*	*D*—H···*A*
O3—H3B···O4^i^	0.82	1.86	2.680 (6)	174
C2—H2A···Cg1^ii^	0.93	2.93	3.722 (5)	144

Symmetry codes: (i) −*x*+1, *y*−1/2, −*z*+3/2; (ii) −*x*, *y*−1/2, −*z*+3/2.
